# Impact of the COVID-19 Pandemic on Higher Education: Characterizing the Psychosocial Context of the Positive and Negative Affective States Using Classification and Regression Trees

**DOI:** 10.3389/fpsyg.2021.714397

**Published:** 2021-09-01

**Authors:** Marina Romeo, Montserrat Yepes-Baldó, Miguel Ángel Soria, Maria Jayme

**Affiliations:** ^1^Department of Social Psychology and Quantitative Psychology, Universitat de Barcelona, Barcelona, Spain; ^2^Department of Clinical Psychology and Psychobiology, Universitat de Barcelona, Barcelona, Spain

**Keywords:** affective states, COVID−19 outbreak, students, teachers and researchers, administrative and services staff, work-life balance, classification and regression trees

## Abstract

Our aim is to analyze the extent to which the psychosocial aspects can characterize the affective states of the teachers, administrative staff, and undergraduate and postgraduate students during the quarantine. A questionnaire was answered by 1,328 people from the community of the *Universitat de Barcelona* (UB), Spain. The survey was partially designed *ad hoc*, collecting indicators related to sociodemographic variables, the impact of COVID on the subjects or in their personal context, the psychosocial context of coexistence and perceived social support, characteristics related to the physical context during the quarantine, and labor conditions. Additionally, it included two validated instruments: the Survey Work-Home Interaction–Nijmegen for Spanish Speaking Countries (SWING-SSC) validated in Spanish and PANAS, the Positive and Negative Affect Schedule. Classification and Regression Trees (CART) were performed to identify which variables better characterize the participants' level of positive and negative affective states. Results according to groups showed that students are the ones who have suffered the most as a result of this situation (temporary employment regulation, higher scores in negative work-home and home-work interaction, lower scores in positive home-work interaction, and negative effects of teleworking). Additionally, they reported a higher mean score in interpersonal conflict and worse scores with regard to negative affective states. Based on sex, women were the ones whose environment was shown to be more frequently affected by the pandemic and who exhibited more negative effects of teleworking. In general terms, participants with the highest scores in negative affective states were those who perceived an increase in conflict and a high negative effect from work spilling over into their personal lives. On the contrary, participants with the highest levels of positive affective states were those with medium to low levels of negative home-work interaction, over 42.5 years old, and with medium to high levels of positive work-home interaction. Our results aim to help higher education to reflect on the need to adapt to this new reality, since the institutions that keep pace with evolving trends will be able to better attract, retain, and engage all the members of the university community in the years ahead.

## Introduction

On March 14th, 2020, Royal Decree 463/2020 (Legislación consolidada, 2020) declared a state of alarm in Spain due to the health crisis caused by COVID-19. All Spanish universities were plunged into an unprecedented situation involving the suspension of all face-to-face teaching activity and consequently, the transition of all such activity to online mode. Giannini, UNESCO Deputy Director-General for Education, pointed out that “to be frank, we must recognize that we were not prepared for such a scale-out disruption. […] The obstacles are multiple, […] [including] low connectivity and lack of online content” UNESCO.(2020, p. 5).

There is no doubt that this difficult and unprecedented period has had an emotional impact on the whole of society in general (Wang et al., 2020). In this regard, several investigations have pointed out that “the greatest impact of an epidemic on mental health is the increase in anxiety, panic, anger, disappointment, sleep problems, disturbances in circadian rhythms, as well as depressive and post-traumatic stress symptoms” (Gismero-González et al., 2020, p. 6). Nevertheless, although the pandemic has affected the entire world, not all work activities have suffered its consequences in the same way. As pointed out by Bania and Banerjee (2020), universities across the globe have responded to COVID-19 in different ways; some have continued face-to-face teaching with social distancing (e.g., the University of Queensland, Australia), while others have deferred all academic activities (e.g., the University of Hyderabad, India), or even moved online (e.g., Harvard University and Massachusetts Institute of Technology, MIT, United States). This last measure was adopted by the majority of universities around the world (67%), and despite this change, as indicated by the report Impact of COVID-19 on Higher Education around the world (Marioni et al., 2020), teaching and learning were not affected. These results demonstrate that Higher Education Institutions (HEIs) had both the technological infrastructure to develop their activities in virtual format as well as the commitment to maintaining the teaching activity on the part of the entire academic community.

Several technical reports, such as those developed by the Ontario Confederation of University Faculty Associations (Ontario Confederation of University Faculty Associations, 2020) and the reports by the European Association for International Education (EAIE), the Erasmus Student Network (ESN), the American Council on Education (ACE), and the International Association of Universities (Marioni et al., 2020), aim to explore the impact of the COVID-19 pandemic on higher education, focusing on education's sudden shift to online teaching and learning and the accompanying opportunities and challenges. Additionally, it is also worth mentioning the research developed by Khoshaim et al. (2020), Sahu (2020), and Zurlo et al. (2020), focusing on the emotional impact of the pandemic on students. However, until now, none of this research has analyzed the psychosocial aspects that characterize the affective states of the entire university community, which includes teachers and researchers, administrative and services staff, and undergraduate and postgraduate students, during the quarantine.

The aim of the paper is to analyze the extent to which the emotional state of the university community members during the quarantine could be predicted by sociodemographic variables, the impact of COVID on the subjects' environment, the psychosocial context of coexistence and perceived social support, characteristics related to the physical context, labor conditions and the work-life balance.

## Method

### Design and Procedure

This cross-sectional research was carried out between May and September 2020 at Universitat de Barcelona. The Equality Unit approved the questionnaire and its administration to the three collectives (teachers and researchers, administrative and services staff, and students) through the University's intranet, once it has been guaranteed by university ethics commission that all the information collected was confidential and was subject to current data protection regulations.

The questionnaire was administered by sending an email invitation and the link to the survey to 72,331 students, teachers and researchers, and administrative and services staff. Participants received a letter ensuring them that all information collected would be confidential and subject to Regulation (EU) 2016/679 of the European Parliament and of the Council of 27th April 2016 (Oficina de Publicaciones de la Unión Europea, 2020) on the protection of individuals with regard to the processing of personal data and the free movement of such data and repealing Directive 95/46/EC (General Data Protection Regulation) (EUR-Lex, 2020). This information was also subject to Organic Law 3/2018, of December 5th, on the protection of personal data and the guarantee of digital rights, which regulates the aspects referred to in the General Data Protection Regulation concerning the national law of each Member State and European data protection legislation (Agencia Estatal Boletín Oficial del Estado, 2018). In addition, as reported in the survey itself, the responses were only used for statistical purposes related to this research and participants were free to withdraw from the study at any point without penalty.

The Universitat de Barcelona (UB), founded in 1450, is one of the oldest universities in Spain. It offers 73 bachelor's degrees, 162 university masters and 48 doctoral programs. It is one of the top five face-to-face universities with the highest number of enrolled students (62,696). It has a staff of more than 8,000 people, 5,825 professors and researchers and 2,864 administration and services staff. In 2020 it is considered the best university in the country in most international rankings such as the Academic Ranking of World Universities developed by Shanghai Ranking, the QS World University Ranking developed by Elsevier, the Best Global Universities developed by the US News & World report, and it occupies reference positions on a European and global scale.

### Participants

The total sample was composed of 1,328 participants, all members of the Universitat de Barcelona (UB), Spain. As to their relationship with UB, 45.8% were students, 29.7% were teachers and researchers (T&R), and 23.8% were administrative and services staff (A&S). This distribution was not equivalent to the study universe, as the number of T&R and A&S was higher than expected, and the number of students was lower [$\chi_{\left(2\right)}^{2}$ = 2,398.27; *p* < 0.001]. Nevertheless, sample errors ranged between 3.96% (for students' sample) and 5.17% (for A&S) with a confidence interval of 95%. The majority were female (69.5%), while men made up 28.2% of participants (1.4% self-identified as non-binary and 0.9% did not answer this question).

In terms of the labor situation during the period under review, 4.5% were employed as health workers, and 4.7% in other essential services. Some of the participants did not work during the pandemic due to various reasons: Record of Temporary Employment Regulation (ERTE, in its Spanish acronym, 2.8%), unemployment (2.4%), retirement (0.6%), paid leave (0.5%), and sick leave (0.5%). A total of 9% had entered the labor market during the pandemic period for the first time, and 25.6% were exclusively studying.

Among respondents who were asked about their job modality (*n* = 799, 60.2%), 20.2% worked exclusively on site, 57.1% performed teleworking, and 22.8% had a blended arrangement. As to their contract status, 36% had permanent contracts, 34.2% held temporary contracts and 29.8% were civil servants. Most of them earned their own incomes (67.5%), although 30.9% were economically dependent and 0.8% declared no income.

When considering the effects of the pandemic on their personal and family working situation, 31.1% affirmed that at least one member of their family had been affected by a record of temporary employment regulation or other employment restrictions. The household economy was negatively affected for 34.1% of the participants. These situations resulted in a high stress level (3.29 on a scale of 1 to 5) for the participants and their families (3.62 on a scale of 1 to 5).

An analysis of the psychosocial context during the quarantine revealed that most of the participants lived with their parents (36.4%), their partner and children (28.8%) or just their partner (15.7%), 6.7% lived alone, 5.9% cohabited with other people (not relatives), and 5.6% were single-parent families. The households were composed of 2–4 adult persons (37.9% 2 persons, 25.5% 3 persons, 18.7% 4 persons) and 0–2 minors (58.7% without minors, 23.8% 1 minor, 12.1% 2 minors). Additionally, 14% lived with at least one elderly person or persons with disabilities, and 42% were responsible for between 1 (21.8%) and 2 persons (20.2%). The score for the perception of increased interpersonal conflict, on a scale of 1–5, was medium-low (*M* = 2.31, *SD* = 1.35).

In terms of the physical context, the majority of participants lived in Barcelona (45.2%) or in the surrounding vicinity (within 50 km) (38.2%). The average size of the residences was 106.95 m^2^, although the median size was 90 m^2^, with considerable positive skewness. The interquartile range was between 70 and 110 m^2^. The majority of residences had a balcony (61.2%) and natural light (85%), and almost half of them had either a terrace, garden, or rooftop terrace (46.5%).

Finally, with reference to the effects of COVID on the participants' context, 65.5% declared that some people in their immediate environment had been affected by the virus and 14.1% had taken a COVID test. Most of the cases reported were mild cases requiring home confinement, although some of the participants had suffered the death of one or more acquaintances (44%), distant relatives (26.8%) or close relatives (14.7%).

### Instruments

The survey was partially designed *ad hoc*, although it was based on two validated instruments: the Survey Work-Home Interaction–Nijmegen for Spanish Speaking Countries (SWING-SSC), validated in Spanish (Romeo et al., 2014), and the Positive and Negative Affect Schedule (PANAS) (Watson et al., 1988).

The Survey Work-Home Interaction–Nijmegen for Spanish Speaking Countries (SWING-SSC) version used in the present study included 11 items, which were answered by each participant scoring on a 5-point Likert scale (1 = strongly disagree and 5 = strongly agree). This instrument evaluates the perceived work-life balance (WLB) by measuring the 4 interactions it describes (Romeo et al., 2014):

- negative work-home interaction (NWHI): negative reactions generated at work that hinder non-work functioning
- negative home-work interaction (NHWI): negative reactions generated in non-work activities that make it difficult to function at work
- positive work-home interaction (PWHI): positive reactions generated at work that facilitate non-work functioning
- positive home-work interaction (PHWI): positive reactions generated in non-work activities that facilitate functioning at work. The reliability of the original version of the instrument was α = 0.80 (Geurts et al., 2005), while the Spanish adaptations were α = 0.83 (Moreno-Jiménez et al., 2009) and α = 0.84 (Romeo et al., 2014).

In the present study, reliability was satisfactory with a global α = 0.76.

The Positive and Negative Affect Schedule (PANAS, Watson et al., 1988; Spanish validation, Sandín et al., 1999) version used in the present study was a self-reported questionnaire containing 15 items for measuring affect according to two independent factors, positive and negative affect. Each item is answered on the basis of a 5-point Likert scale (1 = not at all and 5 = a lot). Positive Affect reflects how energetic and active a person feels, which is associated with social participation and regular physical exercise, while Negative Affect reflects the extent to which a person feels sad low on energy, and predominantly experiences emotions such as guilt, fear, or nervousness, which are related to stressful life events and feelings of anxiety and depression. The PANAS scale, both in its original version and in the Spanish validation, shows adequate psychometric properties: separated by sex, Positive Affect α = 0.89 (men) and 0.87 (women); Negative Affect α = 0.91 (men) and 0.89 (women). In the present study, reliability was satisfactory with a global α = 0.88.

Additionally, we collected indicators related to sociodemographic variables (sex, age, marital status, and university role), the impact of COVID on the subjects or their personal context, the psychosocial context of coexistence and perceived social support, characteristics of the physical context during the quarantine, and labor conditions.

### Data Analysis

All variables under study were described and compared by sex (men and women, as the non-binary category did not include enough individuals), and group (students, T&R, A&S). We described and compared the labor situation and job modality, the negative effects of the pandemic on the labor and economic situation, the psychosocial context and household, the physical context, the effect of COVID on the participants' context, affective states, work-life balance, and the negative effect of teleworking. For comparisons by sex, χ^2^ and *t*-test were used, and for comparison by groups χ^2^ and ANOVA, with Dunnett's C *post-hoc* comparisons, were used.

Secondly, Classification and Regression Trees (CART) using SPSS 25 were performed to identify which variables, when considered simultaneously, better predicted the participants' level of positive and negative affective states. CART, a form of binary recursive partitioning, enables investigators to identify the segments of a diverse population that are most related to a dependent variable (affective states, in this case) based on numerous shared characteristics. We used CART as a statistical approach as it is preferable to other parametric approaches for identifying homogenous subgroups. Additionally, it has greater resistance to the effects of multicollinearity, outliers, and missing data, and is useful for detecting higher-order interactions among predictors before determining which variables should be included in the model (Merkle and Shaffer, 2011).

We obtained the trees with the complete sample, and subsequently split the sample into groups based on sex (men and women) and group (students, T&R, A&S). To avoid overestimation, the level of pruning was fixed at a standard deviation of one. Additionally, the minimum size for the parent node was fixed at 100 for the global sample and 50 for the subsamples, and at 50 and 10 respectively for the child nodes.

## Results

### Descriptive and Comparative Results

#### Labor Situation and Job Modality

Several statistically significant differences were found upon analyzing by sex. The majority of health workers were women (81.7%), and the percentage of women devoted to this job was 6.3%, as opposed to 3.5% for men [$\chi_{\left(1\right)}^{2}$ = 4.06; *p* < 0.05]. The comparison by group also revealed several statistically significant differences. The percentage of students who were exclusively studying, as expected, was significantly higher than that of the other two groups [$\chi_{\left(2\right)}^{2}$ = 681.23; *p* < 0.001]. Of the total number of health workers, 68.1% were students. Additionally, a higher percentage of students were health workers compared to T&R (8.1%, compared to 5.8% of T&R) [$\chi_{\left(2\right)}^{2}$ = 26.07; *p* < 0.001].

With regard to the reports of temporary employment regulation and unemployment, both of these occurred more frequently, almost exclusively, among students (5.6% of the total number of students and 94.4% among all employees were affected by the regulation, and all those unemployed were students).

Statistically significant differences were also observed between groups in terms of job modality [$\chi_{\left(2\right)}^{2}$ = 134.52; *p* < 0.001]. Although the majority of A&S and T&R teleworked, A&S presented higher percentages of the blended arrangement (31.8%). For their part, most of the students who worked (*n* = 136) did so on site (52.9%).

Statistically significant differences were observed between the students and the other two groups in terms of their incorporation into the labor market during the pandemic (11.4% of the students, 8.7% of the A&S and 5.9% of the T&R) [$\chi_{\left(2\right)}^{2}$ = 8.70; *p* < 0.05]. The same was true for the type of contract: most students had temporary contracts (55.2%), while among the A&S, civil servants (45.5%) and permanent contracts (36.5%) prevailed [$\chi_{\left(2\right)}^{2}$ = 132.63; *p* < 0.001]. No statistically significant differences by sex were observed.

In terms of income, no statistically significant differences were observed based on sex, but there were differences according to group, with students being the ones who were most economically dependent on other people (66.4%) [$\chi_{\left(2\right)}^{2}$ = 676.13; *p* < 0.001].

#### Negative Effects of the Pandemic on the Labor and Economic Situation

Again, the students are the ones who have suffered the most as a result of this situation (44.5% suffered a temporary employment regulation report in the family and 67% saw their economic capacity reduced, compared to 24.8–22.7% of A&S and 16.4–19.9% of T&R, respectively) [$\chi_{\left(2\right)}^{2}$ = 95.36; *p* < 0.001]. By sex, no statistically significant differences were observed in terms of the temporary employment regulation report, but there were differences in the degree to which the family economy was affected, with women being the ones who suffered the most in this regard (37.2% of women vs. 26.6% of men) [$\chi_{\left(1\right)}^{2}$ = 13.29; *p* < 0.001].

The stress caused by these labor and economic problems was similar for both men and women [*t*_(423)_ = −1.92, *p* = 0.056], as well as their families [*t*_(428)_ = −1.25, *p* = 0.211]. Conversely, there were statistically significant differences in the family stress perceived by students (M = 3.71) and A&S (M = 3.30), with the former perceiving a greater impact [*F*_(2,435)_ = 4.14, *p* = 0.017].

#### Psychosocial Context and Household

Statistically significant differences were observed according to sex and group. Men and women primarily lived with their parents (39.3 and 35.6%, respectively), or with their partner and children (31 and 28.4%, respectively), whereby these differences were statistically significant [$\chi_{\left(1\right)}^{2}$ = 16.06, *p* = 0.007]. With regard to the groups, the students mainly lived with their parents (73.2%), while A&S and T&R tended to live with their partner and children (45.4 and 56.5%, respectively) [$\chi_{\left(10\right)}^{2}$ = 19.99, *p* = 0.029].

Students (*M* = 3.07, *SD* = 1.113) lived with a greater number of adults than A&S (*M* = 2.39, *SD* = 1.062) and T&R (*M* = 2.39, *SD* = 1.06) [*F*_(2,1,293)_ = 63.20, *p* < 0.001]. In terms of coexistence with minors, the mean for T&R (*M* = 1.05, *SD* = 2.85) was higher than for students (*M* = 0.40; *SD* = 1.41) as well as for A&S (*M* = 0.63; *SD* = 0.85) [*F*_(2,1,288)_ = 14.07, *p* < 0.001]. Lastly, there were statistically significant differences between students (*M* = 0.55; *SD* = 0.90) and A&S (*M* = 1.15; *SD* = 1.21) and students and T&R (*M* = 1.18; *SD* = 1.07) with regard to the number of people in charge, but not between these last two groups [*F*_(2,1,267)_ = 56.17, *p* < 0.001].

As for the perception of increased interpersonal conflict, no statistically significant differences were found based on sex, but there were differences between groups. Specifically, students reported higher mean scores (*M* = 2.57; *SD* = 1.40) than A&S (*M* = 2.08; *SD* = 1.23) and T&R (*M* = 2.08; *SD* = 1.28) [*F*_(2,1,306)_ = 22.22, *p* < 0.001].

#### Physical Context

No statistically significant differences by sex were observed, but there were statistically significant differences according to group [$\chi_{\left(4\right)}^{2}$ = 61.39; *p* < 0.001], with A&S (53.4%) and T&R (54.3%) exhibiting the highest proportion of residences in the city of Barcelona, while most students lived in towns among 10 and 49 km (49.5%) and more than 50 km (22.9%) away from the city. With regard to the size of the residences, there were no statistically significant differences based on sex, but there were indeed differences between groups [*F*_(2,1,262)_ = 3.33, *p* = 0.036]. On average, students (*M* = 111.42 m^2^) and T&R (*M* = 110.04 m^2^) lived in homes that were larger than those of A&S (*M* = 96.66 m^2^).

The percentage of students with balconies in their homes (50.4%) was lower than that of A&S (57.8%) and T&R (53.8%) [$\chi_{\left(2\right)}^{2}$ = 14.93; *p* = 0.001]. Conversely, the percentage of students living in houses with terraces, rooftop terraces and/or gardens (44.7%) was higher than that of A&S (40.9%) but lower than that of T&R (50.8%) [$\chi_{\left(2\right)}^{2}$ = 7.21; *p* = 0.027]. No statistically significant differences were found between the groups with regard to natural light in their homes.

#### The Effects of COVID on the Participants' Context

There were more cases of women whose social context had been affected by the pandemic (69.9% compared to 54.3% of men) [$\chi_{\left(1\right)}^{2}$ = 27.91; *p* < 0.001]. No statistically significant differences were found according to group.

#### Affective States

Although the means obtained from the participants in their positive (*M* = 2.53; *SD* = 0.81) and negative (*M* = 2.39; *SD* = 0.85) affective states were very close, the *t*-test for related samples showed statistically significant differences between both values [t_(1, 306)_ = 3.66, *p* < 0.001], whereby these were higher for the positive effects. This trend is observed for men [*t*_(368)_ = 5.84, *p* < 0.001], A&S [*t*_(306)_ = 11.03, *p* < 0.001], and T&R [*t*_(389)_ = 6.02, *p* < 0.001], while in the case of students, negative effects prevailed [*t*_(605)_ = −6.27, *p* < 0.001]. No statistically significant differences were found for women between the scores for positive and negative effects.

Comparing affective states by sex, statistically significant differences in negative affective states were found [*t*_(1,287)_ = −5.58, *p* < 0.001]. Men exhibited significantly lower scores (*M* = 2.18) than women (*M* = 2.47). Statistically significant differences were also observed between groups, both for positive [*F*_(2,1,302)_ = 53.40, *p* < 0.001] and negative affective states [*F*_(2,1,307)_ = 59.56, *p* < 0.001]. Multiple comparisons using Dunnet's C indicated the existence of statistically significant differences between all groups in terms of positive affective states, whereby A&S was the group with the highest scores, followed by T&R, with students in last place. As for negative affective states, statistically significant differences were only found between students and the other two groups, with no differences between A&S and T&R. Again, in this case, the students produced worse scores, since their average for negative effects was higher than that of the other groups.

#### Work-Life Balance

Comparisons of means obtained from related samples indicated the existence of statistically significant differences between all subdimensions [NWH-NHW: *t*_(1,308)_ = 30.67, *p* < 0.001; NWH-PWH: *t*_(1,308)_ = 10.82, *p* < 0.001; NWH-PHW: *t*_(1,296)_ = 12.51, *p* < 0.001; NHW-PWH: *t*_(1,303)_ = −14.22, *p* < 0.001; NHW-PHW: *t*_(1,296)_ = −8.99, *p* < 0.001; PWH-PHW: *t*_(1,293)_ = 4.26, *p* < 0.001]. The highest score was obtained from the negative effect exerted by work on the person's life (NWH), while the lowest score came from the inverse context of life on work (NHW).

Based on sex, statistically significant differences were observed between the four WLB subdimensions. For women, these were the same as the overall sample [NWH-NHW: *t*_(915)_ = 36.64, *p* < 0.001; NWH-PWH: *t*_(910)_ = 9.32, *p* < 0.001; NWH-PHW: *t*_(907)_ = 10.74, *p* < 0.001; NHW-PWH: *t*_(910)_ = −12.23, *p* < 0.001; NHW-PHW: *t*_(907)_ = −7.97, *p* < 0.001; PWH-PHW: *t*_(904)_ = 3.51, *p* < 0.001]. For men, statistically significant differences were only observed between the negative subdimensions [NWH-NHW: *t*_(368)_ = 15.81, *p* < 0.001; NWH-PWH: *t*_(369)_ = 5.33, *p* < 0.001; NWH-PHW: *t*_(365)_ = 5.90, *p* < 0.001; NHW-PWH: *t*_(368)_ = −7.79, *p* < 0.001; NHW-PHW: *t*_(365)_ = −5.00, *p* < 0.001; PWH-PHW: *t*_(365)_ = 1.91, *p* = 0.057].

Similarly, in the analysis based on group, it was observed that for students [NWH-NHW: *t*_(604)_ = 21.52, *p* < 0.001; NWH-PWH: *t*_(602)_ = 13.96, *p* < 0.001; NWH-PHW: *t*_(601)_ = 14.29, *p* < 0.001; NHW-PWH: *t*_(602)_ = −4.77, *p* < 0.001; NHW-PHW: *t*_(601)_ = −2.60, *p* < 0.001; PWH-PHW: *t*_(599)_ = 2.21, *p* = 0.027] and T&R [NWH-NHW: *t*_(390)_ = 19.81, *p* < 0.001; NWH-PWH: *t*_(389)_ = 6.16, *p* < 0.001; NWH-PHW: *t*_(383)_ = 9.24, *p* < 0.001; NHW-PWH: *t*_(388)_ = −10.46, *p* < 0.001; NHW-PHW: *t*_(383)_ = −4.27, *p* < 0.001; PWH-PHW: *t*_(382)_ = 5.81, *p* < 0.001], statistically significant differences were again present between all subdimensions, while for A&S, there were no statistically significant differences between PWHI and PHWI [NWH-NHW: *t*_(308)_ = 11.31, *p* < 0.001; NWH-PWH: *t*_(307)_ = −3.77, *p* < 0.001; NWH-PHW: *t*_(306)_ = −3.95, *p* < 0.001; NHW-PWH: *t*_(307)_ = −12.45, *p* < 0.001; NHW-PHW: *t*_(306)_ = −10.93, *p* < 0.001; PWH-PHW: *t*_(306)_ = −0.79, *p* = 0.432].

Upon analyzing the scores in each subdimension by sex, statistically significant differences were observed in the four subdimensions. Specifically, women demonstrated higher scores than men in all dimensions [NWHI: *t*_(1,287)_ = −4.34, *p* < 0.001; NHWI: *t*_(736.67)_ = −2.59, *p* = 0.01; PWHI: *t*_(1,279)_ = −3.90, *p* < 0.001; PHWI: *t*_(1,273)_ = −3.04, *p* = 0.002].

When comparing the various groups, statistically significant differences were observed in NWHI [*F*_(2,1,308)_ = 110.70, *p* < 0.001], NHWI [*F*_(2,1,304)_ = 37.64, *p* < 0.001], and PHWI [*F*_(2,1,993)_ = 7.31, *p* = 0.001], but not in PWHI [*F*_(2,1,300)_ = 0.37, *p* = 0.692]. Dunnett's *post-hoc C*-test indicated that students had higher scores than A&S and T&R in NWHI and NHWI, and lower than T&R in PHWI. For their part, T&R and A&S differed in the three indicated subdimensions, whereby T&R was the group with the lowest scores in PHWI and the highest in NWHI and NHWI.

#### Negative Effect of Teleworking/Telestudying

Lastly, the negative effect of teleworking on the participants was analyzed. The mean score obtained for the entire sample was 3.45 (*SD* = 1.04) on a scale of 1 to 5, indicating medium-high levels of negative effects as a result of teleworking or “telestudying.” Specifically, the most common negative effects were working outside of working hours or the study schedule (*M* = 3.93, *SD* = 1.27), feeling permanently “on call” due to work/study demands (*M* = 3.66, *SD* = 1.30), and the perception that the use of technology meant that they spent more time studying or working, causing feelings of stress (*M* = 3.43, *SD* = 1.35). In turn, with regard to the statement that working or studying with ICTs led to discomfort, irritability, and impatience, the participants' scores were in the middle range (*M* = 2.77, *SD* = 1.37).

Women presented slightly more negative effects of teleworking [t_(1,288)_ = −2.06, *p* = 0.04], with a mean of 3.49 (*SD* = 1.03), compared to the mean of 3.36 for men (*SD* = 1.05). By group, some statistically significant differences were observed [*F*_(2,1,309)_ = 60.39, *p* < 0.001]; specifically, between students and A&S, and between A&S and T&R, whereby A&S were less affected byteleworking.

#### Effects of the Pandemic on Eating and Sleeping Habits

In global terms, the pandemic had a moderate effect on eating (M = 2.41; SD = 0.85) and sleeping habits (M = 2.78; SD = 0.94). Based on sex, some statistically significant differences were observed, whereby women were more affected in both habits [eating: *t*_(1,288)_ = −2.45, *p* = 0.014; sleeping: *t*_(1,288)_ = −2.32, *p* = 0.021]. In terms of groups, statistically significant differences were also evident [eating: *F*_(2,1,309)_ = 22.34, *p* < 0.001; sleeping: *F*_(2,1,309)_ = 30.77, *p* < 0.001]. Dunnet's C comparisons showed that students were the most greatly affected, ahead of A&S and T&R, with these two groups exhibiting equal levels.

### Classification Trees (CART) for the Negative Affective States

The decision trees generated with the aim of characterizing negative affective states are presented below, both at a general level with the entire sample and by segmentation variables (sex and group) (Table 1).

**Table 1:** Description of the trees for the negative affective states.

	**General sample**	**Sex**		**Group**		
		**Male**	**Female**	**Students**	**A&S**	**T&R**
Total nodes	12	15	27	13	11	7
Depth	5	5	5	4	3	2
Terminal nodes	7	8	14	7	6	4
Initial variance	0.73	0.68	0.72	0.71	0.60	0.66
Risk	0.48	0.38	0.42	0.49	0.39	0.43
Explained variance	34.9%	43.74%	42.01%	31.35%	35.57%	34.65%

#### Predictive Variables of Negative Affective States for the Global Sample

**Figure 1:**
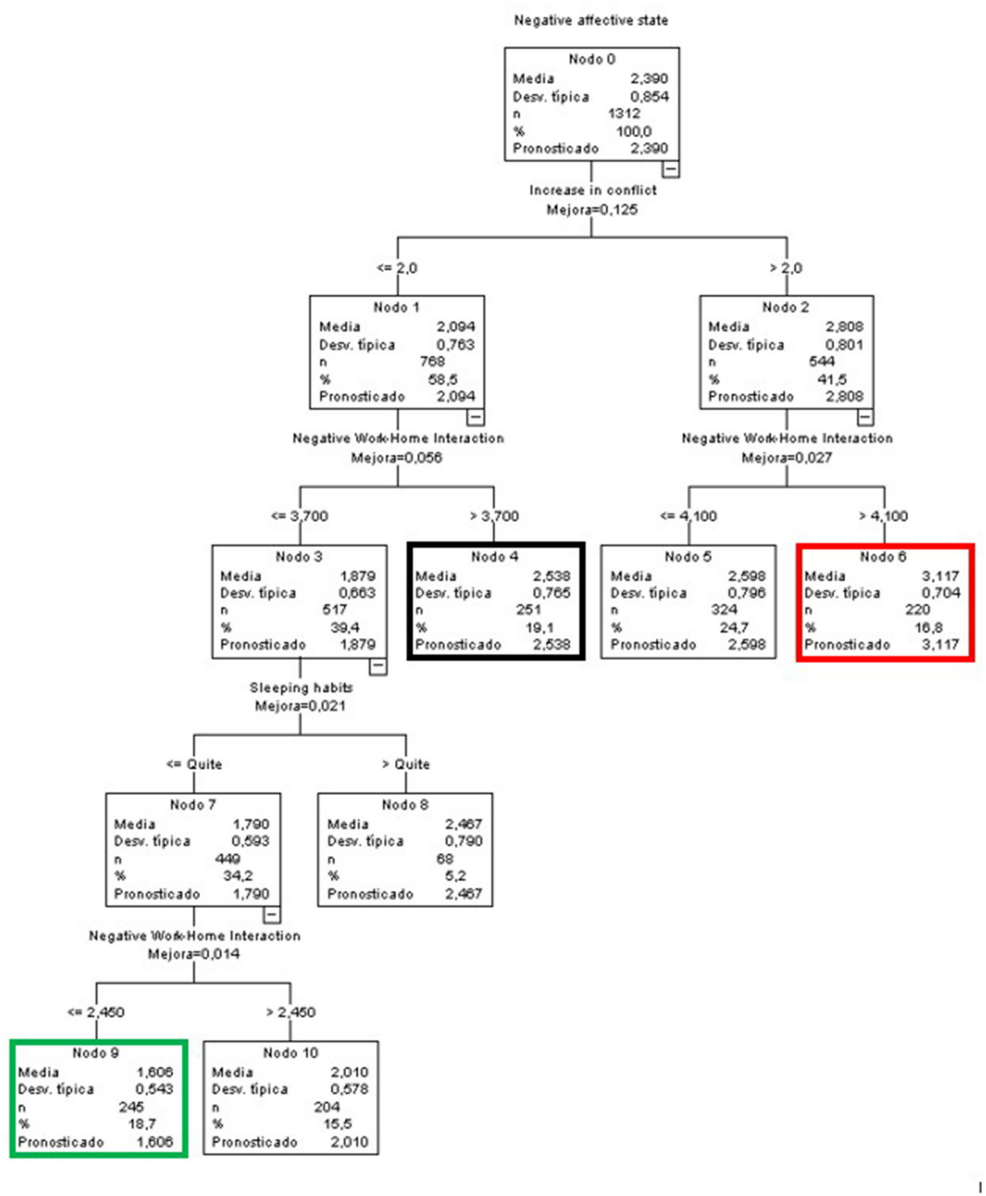
Classificatory tree of negative affective states (Complete sample).

The first variable that is introduced is the increase in conflict, followed by the NWHI. The following branch includes 2 different variables related to the effects of COVID on healthy habits, i.e., sleep and diet, respectively. Finally, the NWHI reappears in the last branch of the model (Figure 1). In the figures, we used colors to indicate the node with the best score (green), the worst score (red), and the largest node in terms of number of participants included (black).

The node with the lowest predicted value in negative affective states is number 11 (green node), with a mean more than 0.7 points lower than the mean of the initial node. In other words, the people in this node had a very low mean score in negative affective states compared to the overall mean of the sample. This group (18.7% of the sample) included people who did not perceive an increase in conflict (scores ≤ 2 on a scale of 1 to 5), whose sleeping and resting habits were more or less affected, and who did not feel that their work interfered with their family life (NWHI ≤ 2.45).

At the other extreme, we find node 6 (red node), with a mean 0.7 points higher than the overall mean, indicating medium-high levels of negative affective states. The participants in this node represented 16.8% of the sample and perceived an increase in conflict above 2 and a high negative impact of their work on their life (NWHI) (above 4.1).

Finally, node 4 (black node) had the highest percentage of participants among all terminal nodes (19.4%). The members of this group were characterized by a score slightly higher than the average; they did not perceive an increase in interpersonal conflict (scores equal to or lower than 2) yet they did perceive a negative effect of their work on their personal life (NWHI) (scores above 3.7).

#### Predictive Variables of Negative Affective States by Sex

##### Men

**Figure 2:**
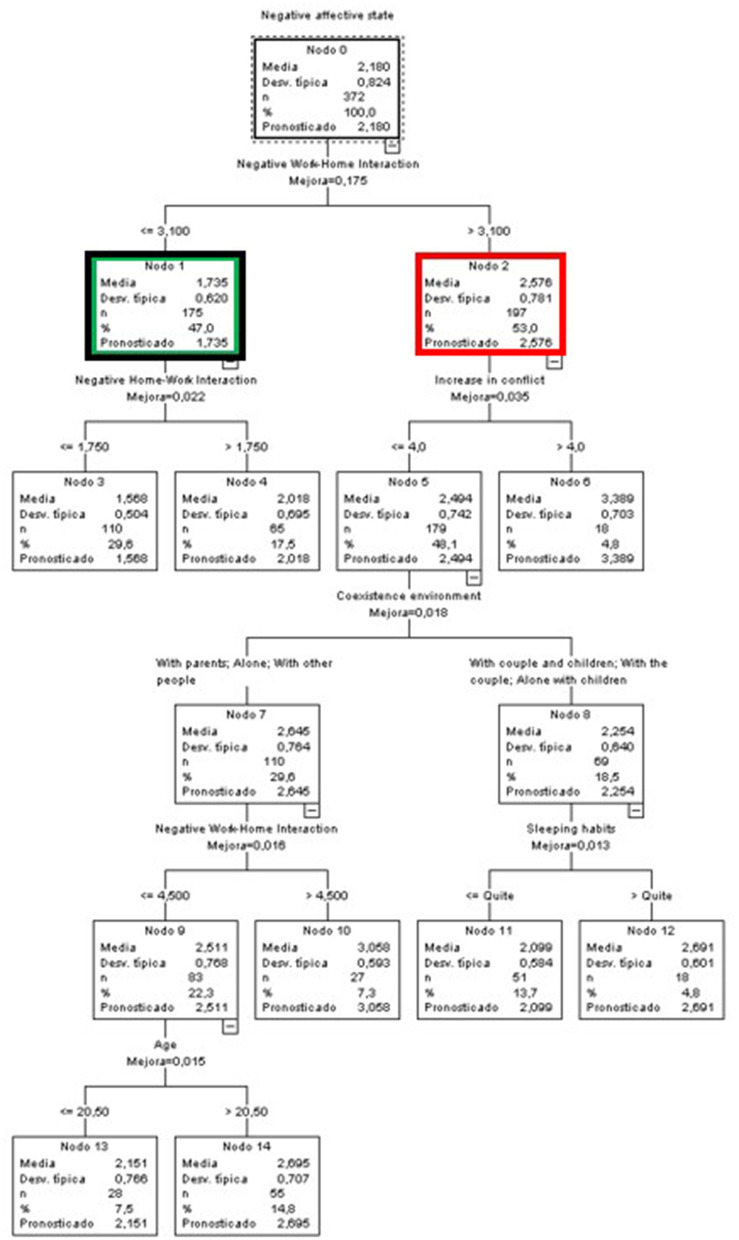
Classificatory tree of negative affective states (Men).

For the group consisting of men (Figure 2), the variables that best predicted negative affective states were again the WLB (negative aspects), an increase in conflict, the coexistence environment, the impact on sleeping and resting habits, and in addition, age now also appears as a new predictive variable.

The node that displayed a lower mean compared to the group mean was node 3 (*M* = 1.568) (green node). In this group, 29.6% of men had the lowest scores in negative affective states, characterized by perceiving a negative effect of work on family life (NWHI) lower than 3.1 and a negative effect of personal life on work (NHWI) lower than or equal to 1.75. Furthermore, this terminal node had the highest percentage of participants (black node). At the opposite extreme we had 4.8% of the men grouped in node 6 (red node), who perceived a negative impact of work on their personal life (NWHI) (above 3.1) and an increase in interpersonal conflict (above 4).

##### Women

**Figure 3:**
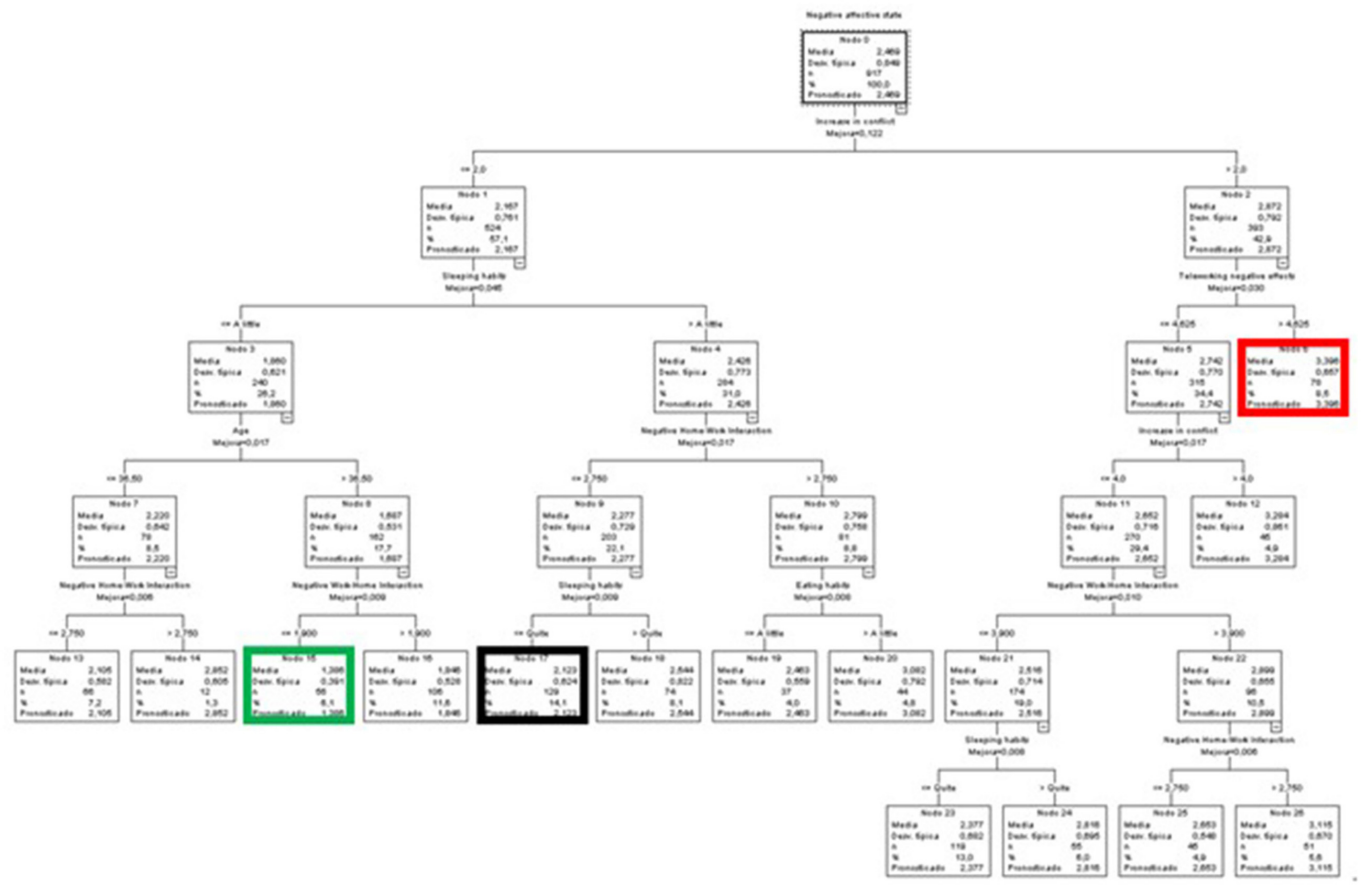
Classificatory tree of negative affective states (Women).

In the case of the women, the tree became more complex, reaching 27 nodes and 5 levels. In addition, although the WLB (in its negative aspect) and age continued to appear as predictive variables, as was the case for the men, new variables also emerged, such as the negative effects of teleworking and the effect of COVID on eating habits. In addition, the foremost predictive variable was the increase in conflict, whereas for men it was NWHI (Figure 3).

The node that reported the lowest mean compared to the group was node 15 (green node). In this group, 6.1% of the women had the lowest scores in negative affective states. They were characterized by perceiving no or barely any increase in conflict (scores ≤ 2), noticing little or no change in their sleeping and resting habits, being over 36.5 years old, and perceiving no or little negative impact of work on their life (NWHI) (scores ≤ 1.9).

At the opposite extreme were 8.5% of women with higher scores in negative affective states, grouped in node 6 (red node), who perceived an increase in conflict (>2) and in the negative effect of teleworking (above 4.625).

Finally, the terminal node with the highest percentage of participants (14.1%) was node 17 (black node). In this case, the mean score for negative affective states was slightly lower than the general mean (*M* = 2.123). This node included women who did not perceive an increase in conflict (scores ≤ 2), whose sleep habits were affected only moderately (between a little and a lot), and who did not perceive a negative effect of work on their personal life (NWHI) (scores ≤ 2.75).

#### Predictive Variables of Negative Affective States by Group

##### Students

**Figure 4:**
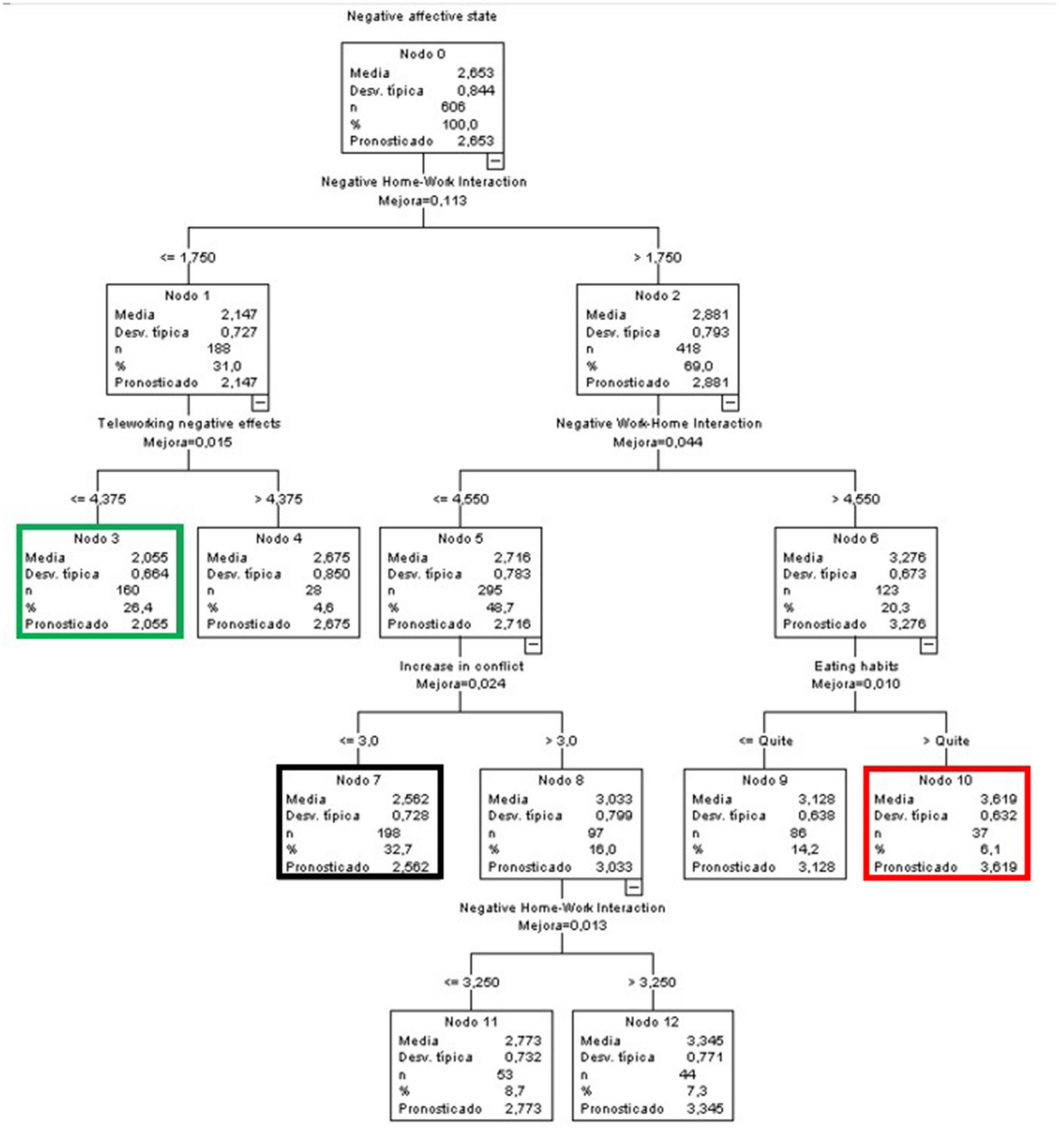
Classificatory tree of negative affective states (Students).

The results for the students' group are shown first. Once again, the WLB was the main predictive variable, but in this case, the first dimension that appeared in the tree was the negative effect of life on work (NHWI). The other variables included were NWHI, negative effects of teleworking (or “telestudy”), the increase in conflict and the effect on eating habits (Figure 4).

Node 3 reported the lowest average score (green node), and therefore the most favorable, with 26.4% of the students and with an average 0.6 points lower than the global average of the group (*M* = 2.055). This node included the students who did not perceive a negative interference of their life in their work (NHWI) (scores equal to or lower than 1.75) and who perceived the negative effects of the “telestudy” to be below or equal to 4.375.

The highest average appeared in node 10 (red node), with 6.1% of the participants of this group and an average of 3.619, almost one point above the general average. This node contained the students most affected by NHWI (above 1.75) and NWHI (above 4.55), and with eating habits that were considerably or severely affected.

Finally, the node with the most participants was 7 (32.7% of the total) (black node). This node had a mean slightly lower than the general one and included students with NHWI above 1.75, NWHI equal to or lower than 4.55 and who perceived little or no increase in conflict (scores equal to or lower than 3).

##### Administrative and Service Staff

**Figure 5:**
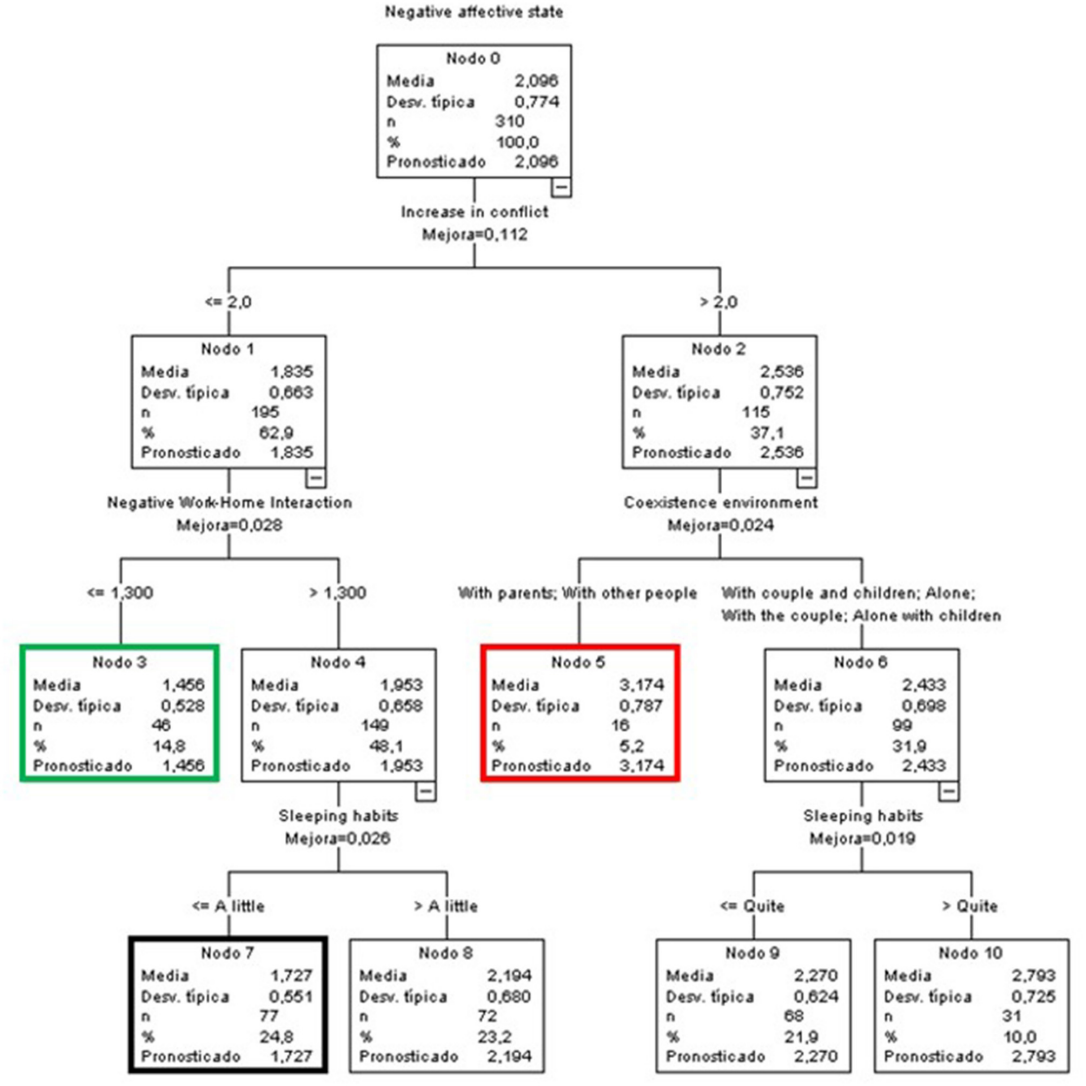
Classificatory tree of negative affective states (A&S).

In this case, the first predictive variable was the increase in conflict; other variables that contributed to the model were NWHI, the coexistence environment, and the effect on sleeping and resting habits (Figure 5).

The node with the lowest mean score in negative affective states among the A&S group was node 3 (*M* = 1.456) (green node), at 0.64 points below the general mean. It included 14.8% of this group and was comprised of people who did not perceive an increase in conflict (scores ≤ 2) or negative interference of work on life (NWHI) (scores ≤ 1.3).

At the other extreme, node 5 (red node) presented the highest mean (*M* = 3.174), more than one point above the general mean, and included 5.2% of the A&S members. It was characterized by people who perceived higher levels of an increase in conflict (above 2) and who lived with their parents or people other than their partners and children.

Finally, node 7 (black node) included the highest percentage of members of the group (24.8%). Its members had an average score of 1.727 (0.37 points lower than the general mean) and were characterized by not perceiving an increase in conflict (scores ≤ 2), an NWHI effect >1.3, and little or no impact on their sleeping and resting habits.

##### Teachers and Researchers

**Figure 6:**
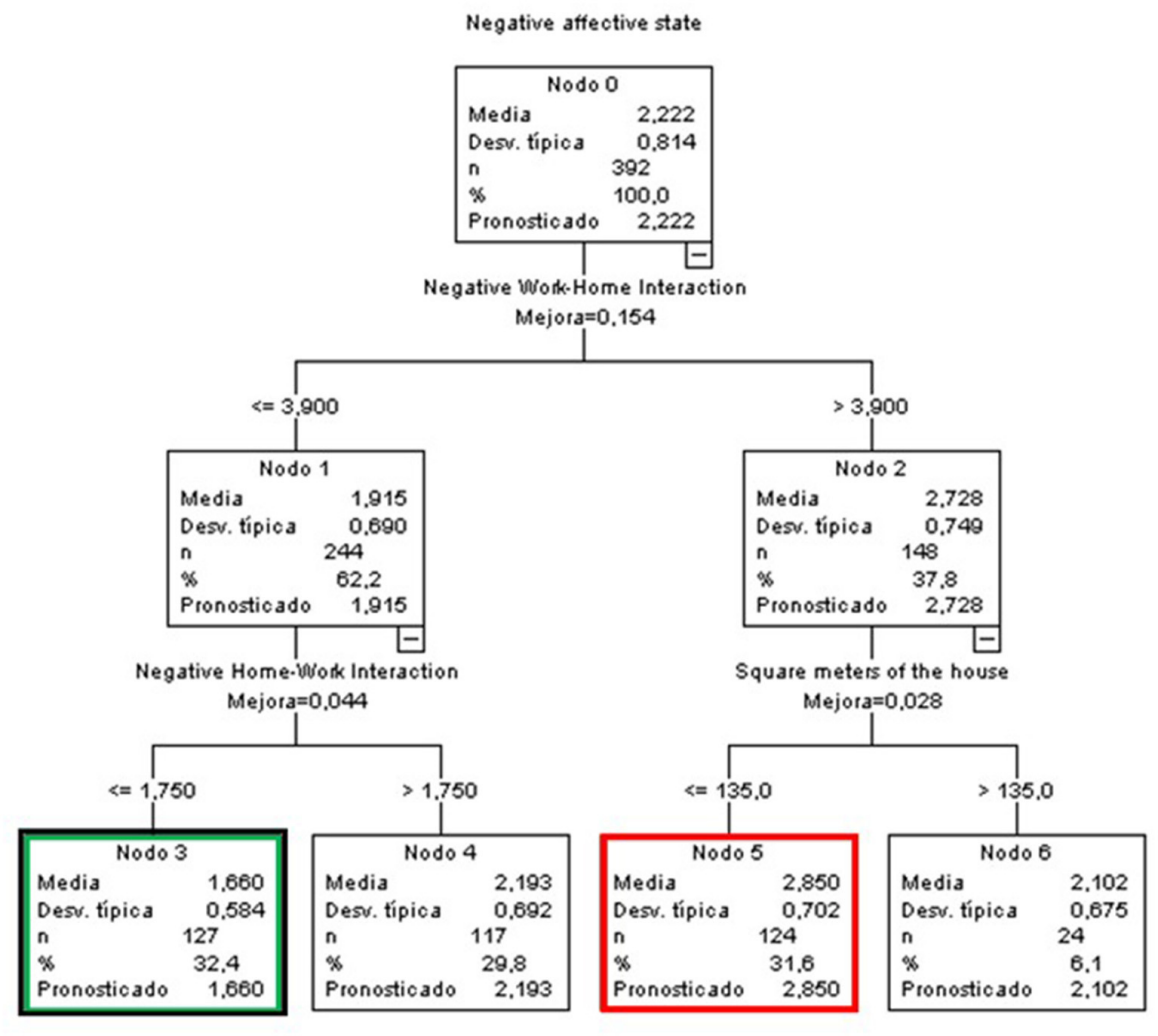
Classificatory tree of negative affective states (T&R).

Finally, in the case of T&R, the foremost predictive variable was the NWHI, accompanied by NHWI and a new variable “residence size” (Figure 6). In this case, the same node, number 3 (green and black node), grouped together the participants with the lowest score (*M* = 1.66, 0.562 points below the general mean) and included the highest percentage of participants (32.4%). This node was characterized by T&R who perceived less interference from their work on their life (NWHI) (≤ 3.9) as well as from their life on their work (NHWI) (≤ 1.75).

On the opposite end of the spectrum, we find node 5 (red node), with a mean of 2.85 (0.628 points above the global mean), which grouped together 31.6% of the sample. In this case, the participants were characterized by high levels of NWHI interference (above 3.9) and by living in residences of 135 m^2^ or less.

### Classification Trees (CART) for the Positive Affective States

The decision trees generated to characterize positive affective states are presented below, both at a general level, with the entire sample, and by segmentation variables (sex and group) (Table 2).

**Table 2:** Description of the trees for positive affective states (PAS).

	**Global sample**	**Sex**		**Group**		
		**Male**	**Female**	**Students**	**A&S**	**T&R**
Total nodes	18	13	25	17	17	11
Depth	5	4	5	4	5	4
Terminal nodes	10	7	13	9	9	6
Initial variance	0.66	0.68	0.65	0.62	0.55	0.64
Risk	0.49	0.49	0.45	0.46	0.40	0.50
Explained variance	24.71%	27.36%	30.83%	26.16%	27.12%	21.60%

#### Predictive Variables of Positive Affective States for the Global Sample

**Figure 7:**
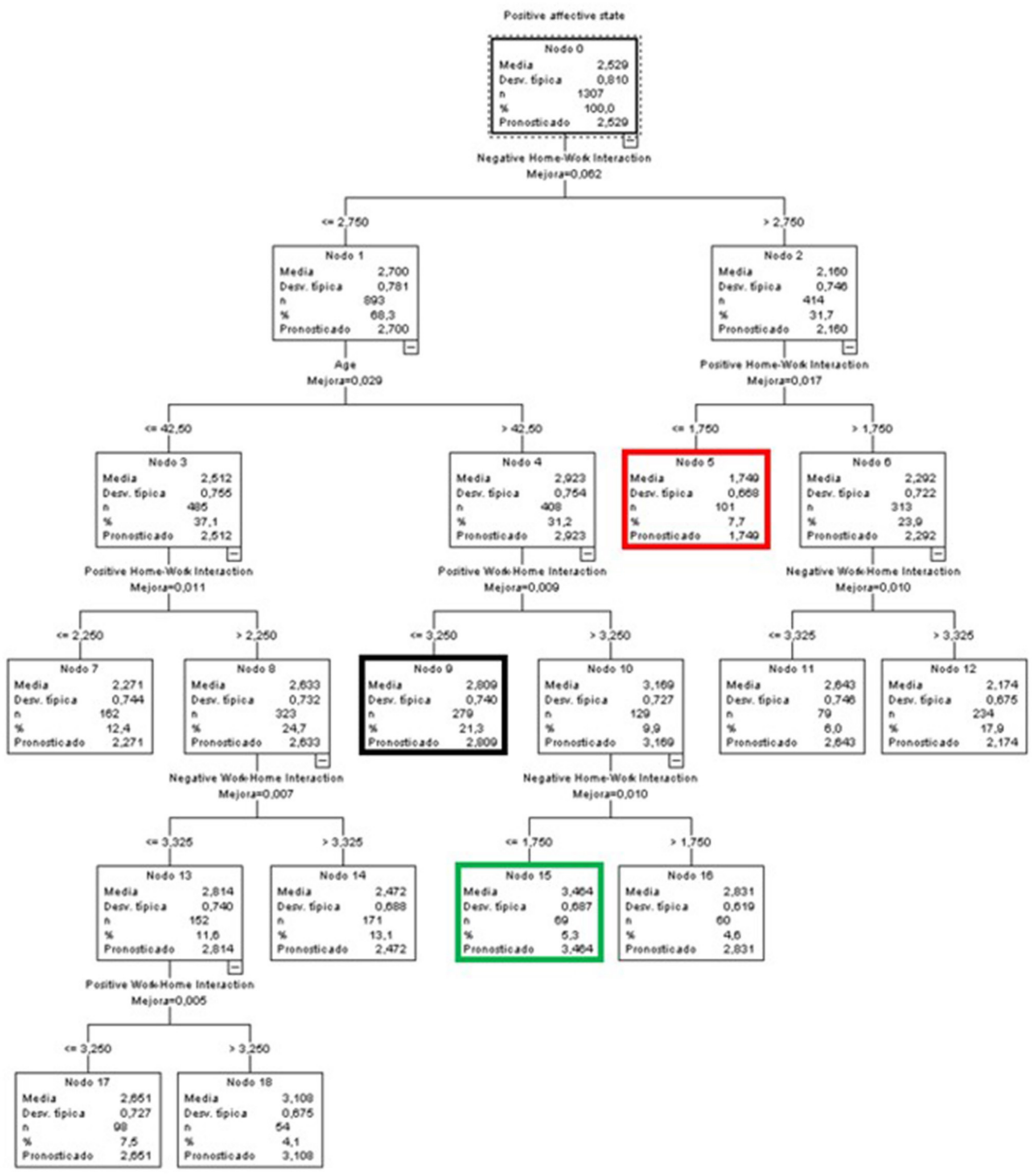
Classificatory tree of positive affective states (Complete sample).

In the case of the predictive model of positive affective states for the entire sample (Figure 7), the variable that almost fully explained the observed variance by itself was the WLB in all its aspects, both positive and negative, and both HWI and WHI. Furthermore, the age variable also influenced the first branch (Node 1). This node included participants with low levels (≤ 2.75) in NHWI. The age factor established two branches, dividing the sample into people below and people above 42.5 years of age.

The terminal node with the highest score in the positive affective state was node 15 (green node), with an average of 3.464, almost one point above the global average. This group was characterized by low NHWI levels (equal to or below 1.75), people aged over 42.5 years, and high PWHI levels (above 3.25).

At the opposite end were people in node 5 (red node), which included 7.7% of the participants in the sample. These were people with NWHI levels >2.75 and low PHWI levels (≤ 1.75).

Finally, node 3 (black node) included the highest percentage of cases overall (21.3%). Its mean was 0.028 points higher than the general mean, and included people with low levels of NHWI (equal to or below 2.75), aged over 42.5 years, and with low levels of PWHI (≤ 3.25).

#### Predictive Variables of Positive Affective States by Sex

##### Men

The node with the highest score was node 4 (green and black node), with an average of 2.987 (0.408 points above the general score) bringing together 33.9% of the group, making it the node with the highest percentage. It was made up of men who perceived a medium to low NWH interference (≤ 3.9) and a high PHWI (above 2.75).

**Figure 8:**
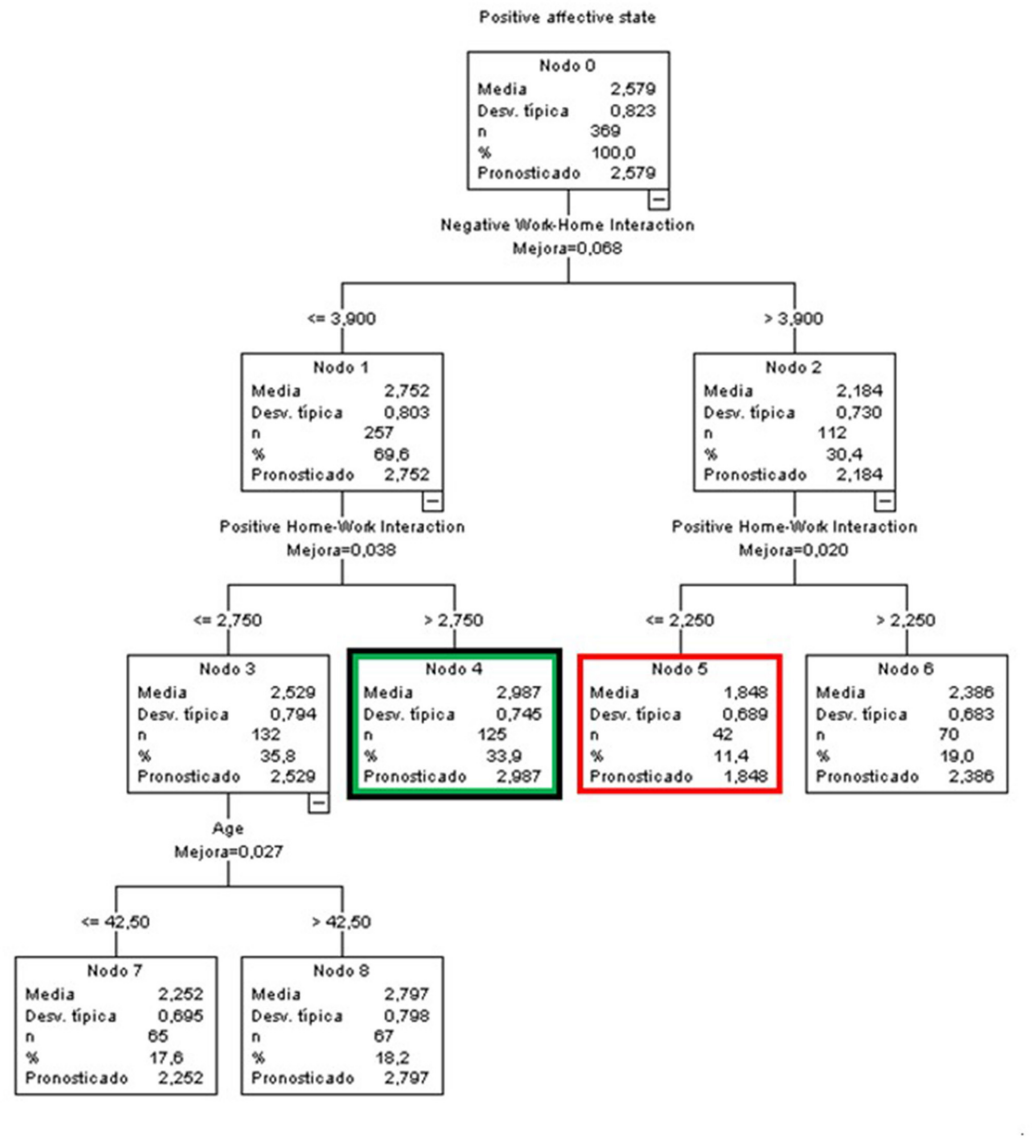
Classificatory tree of positive affective states (Men).

On the contrary, node 5 (red node), with a mean of 1.484 (1.095 points below the global mean), included 11.4% of the sample of men, demonstrating the lowest score in positive affective states, a high NWH interference (above 3.9) and a low PHWI (equal to or below 2.25) (Figure 8).

##### Women

**Figure 9:**
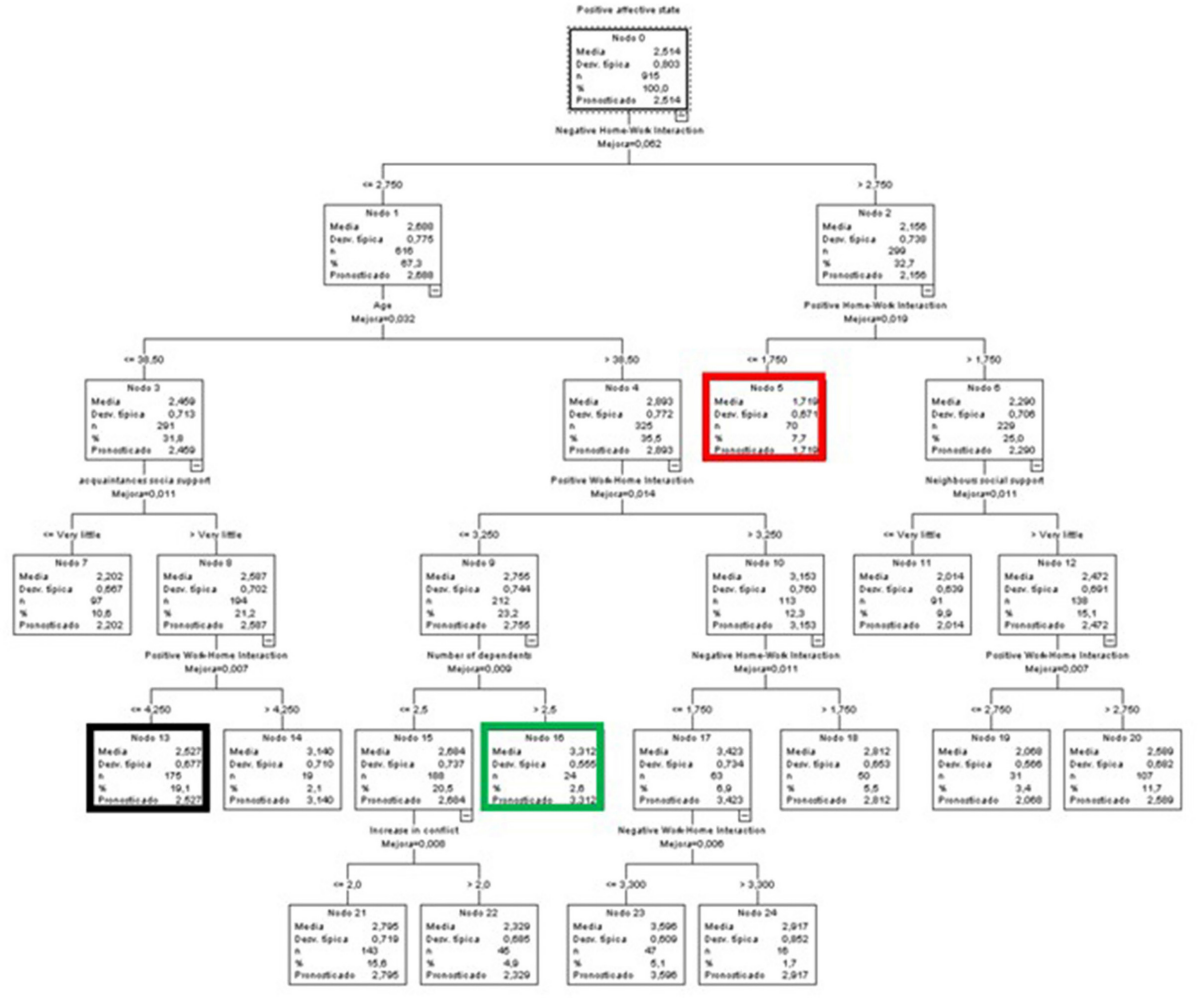
Classificatory tree of positive affective states (Women).

In the case of women, a more complex tree was generated (Figure 9). The highest score was found in node 16 (green node), with a mean of 3.312 (0.798 points above the global mean). It included 2.6% of the sample of women and was characterized by low levels of NHWI (≤ 2.75), those aged over 38.5 years, with medium to low levels of PWHI (≤ 3.25) and with more than 2.5 dependent persons under their responsibility.

Conversely, women with the worst score in positive affective states (1.719) were found in node 5 (red node) and represented 7.7% of the total. In this case, they were characterized by medium to high levels of NWHI (above 2.75) and very low levels of PHWI (equal to or below 1.75).

Finally, the node with the highest number of participants (19.1%, node 13, black node) had a mean slightly higher than the global one (2.527, i.e., 0.013 points above the global mean). It was characterized by women with medium to low NWHI scores (≤ 2.75), aged 38.5 years or younger, who perceived some support from acquaintances (very little to a lot) and with PWHI levels ≤ 4.25.

#### Predictive Variables of Positive Affective States by Group

##### Students

**Figure 10:**
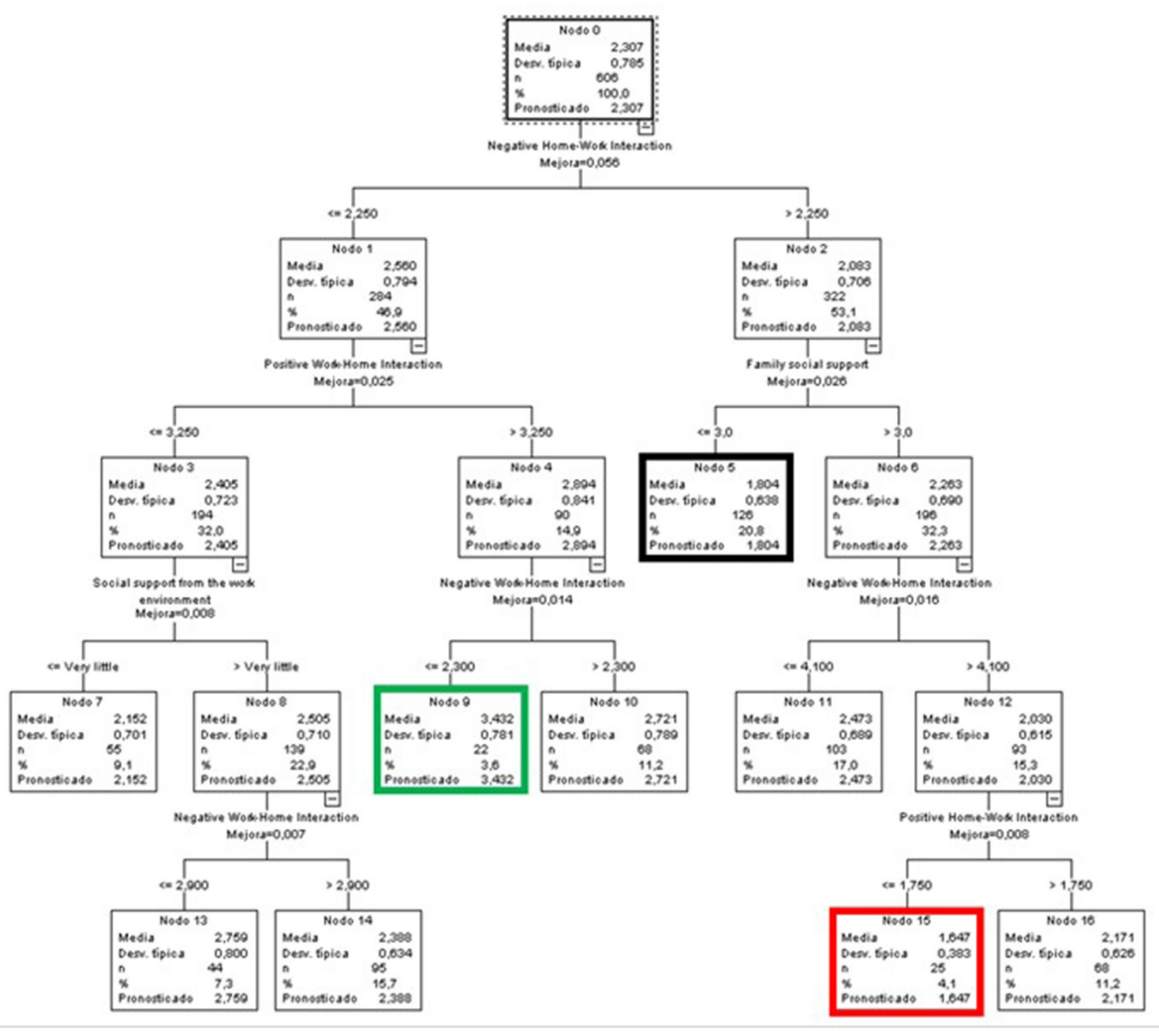
Classificatory tree of positive affective states (Students).

The predictive variables were the WLB (in all its variants) and the perceived support from family and the work/academic context (Figure 10).

The node with the highest score was node 9 (green node), with a mean of 3.432 (1.125 points above the global mean) and 3.6% of the student sample. This included students with low levels of NHWI (≤ 2.25), high scores in PWHI (>3.25) and low scores in NWHI (≤ 2.3).

The lowest mean was found at node 15 (red node) (1.647 i.e., 0.66 points below the global mean), and included 4.1% of the sample. It was characterized by students with medium to high levels of NHWI (above 2.25), high levels of family support (above 3), high levels of NWHI (above 4.1), and very low levels of PHWI (equal to or below 1.75).

Finally, node 5 (black node) included the highest percentage of participants (20.8%), characterized by medium to high levels of NHWI (above 2.25) and medium to low family support (≤ 3).

##### Administrative and Services Staff

**Figure 11:**
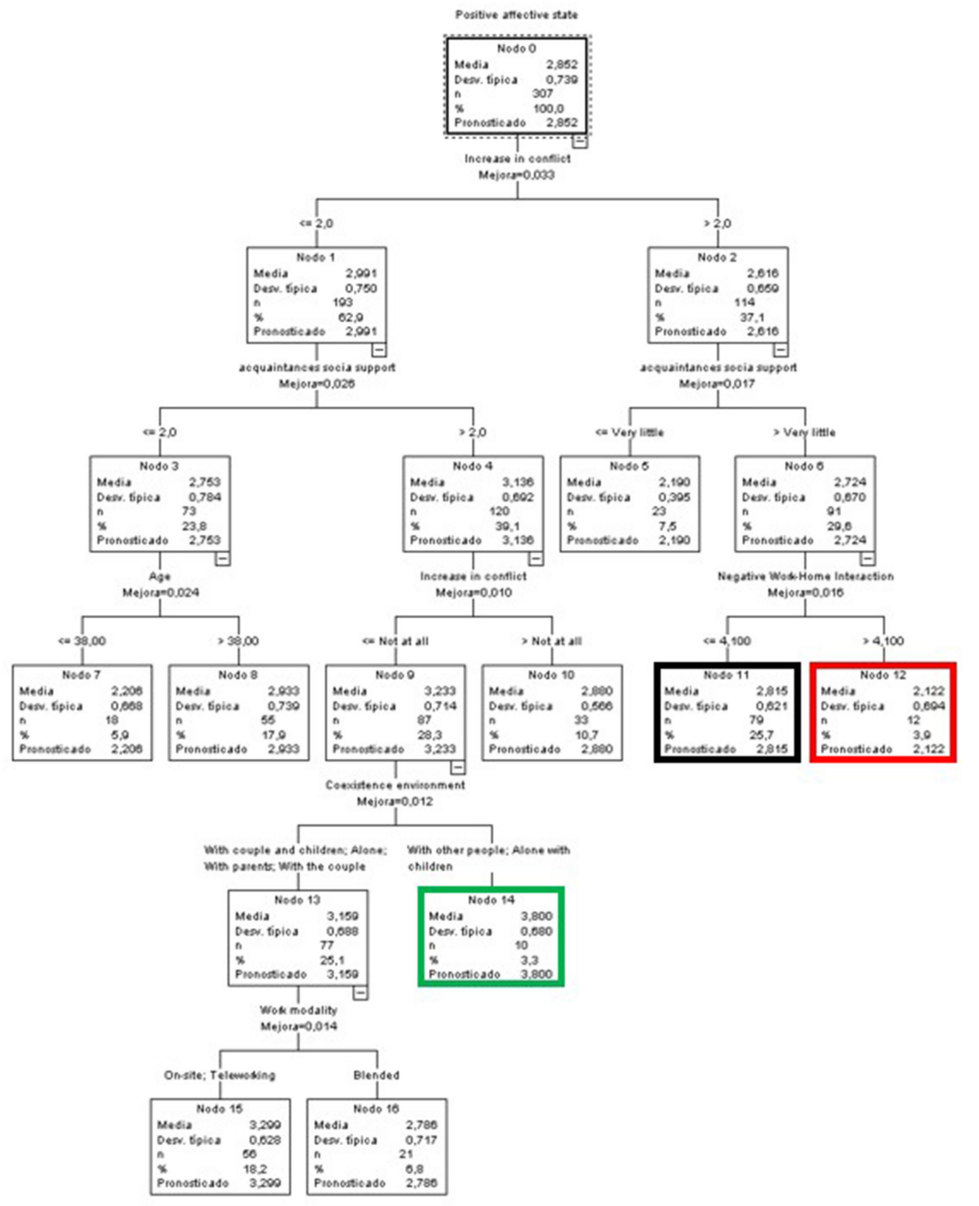
Classificatory tree of positive affective states (A&S).

The predictive variables included in the model for A&S were an increase in conflict (as the foremost variable), support from acquaintances, age, NWHI, the coexistence environment and the modality of work (on-site, teleworking, or blended arrangement) (Figure 11).

The node with the highest mean was node 14 (green node), with 3.3% of participants. The average (3.8) was 0.948 points above the global average for this group. It was characterized by people who perceived little increase in conflict (≤ 1), medium to high support from acquaintances (>2), and who lived alone with their children or other people (who were not their parents or partners).

At the other extreme was node 12 (red node), with a mean of 2.12 (0.732 points below the general mean), which included 3.9% of the A&S sample. It was characterized by increases in conflict and acquaintance support levels above 2, as well as high NWHI levels (above 4.1). Finally, node 11 (black node) included the highest percentage of the sample (25.7%) and was characterized by increases in conflict and a level of support from acquaintances above 2, and NWH levels equal to or below 4.1.

##### Teachers and Researchers

**Figure 12:**
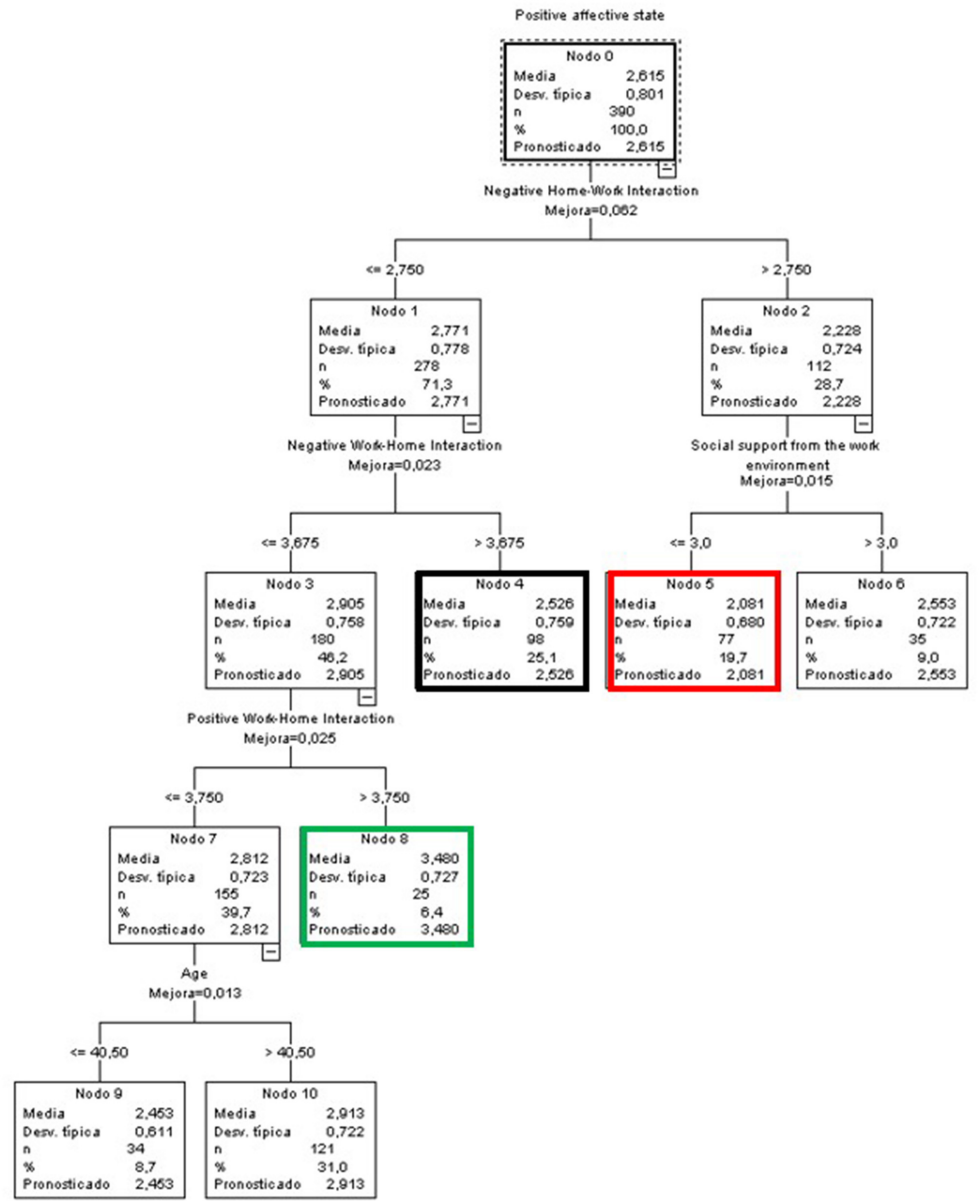
Classificatory tree of positive affective states (T&R).

The tree generated for T&R included the variables NWHI and PWHI, perceived support at work, and age (Figure 12). Node 8 (green node) exhibited the highest mean (3.48), which was 0.865 points above the general mean, and included 6.4% of the T&R sample. It included people with low levels of NHWI (≤ 2.75), medium to low levels of NWHI (≤ 3.675), and high levels of PWHI (above 3.75).

Node 5 (red node), on the other hand, had a mean of 2.081 (0.534 points below the global PDI) and included 19.7% of this group. It was characterized by medium to high levels of NHWI (above 2.75) and medium to low levels of perceived support at work (≤ 3).

Finally, the node with the highest percentage of cases was node 4 (black node), at 25.1%, with a mean slightly lower than the overall T&R sample (2.526 i.e., 0.089 points below the global mean for T&R). The members of this node displayed medium to low levels of NHWI (≤ 2.75) and high levels of NWHI (>3.675).

### Summary of CART Results

The results obtained using CART in our research provide us with specific insight into the psychosocial factors that characterized the affective states of the university community during the quarantine. In general terms, as shown in the trees, there were two main causes of affective states: (a) the perception of increase in conflict, and (b) the work-life balance. Table 3 summarizes the main results, illustrating the groups of participants with the highest scores in negative and positive affective states.

**Table 3:** Comparison of groups with the highest scores in affective states.

			**Negative affective states highest score**	**Positive affective states highest score**
Global sample		Mean	3.117	3.464
		Predictors	Increase in conflict > 2 NWHI > 4.1	NHWI ≤ 2.75 Age > 42.5 PWHI > 3.25 NHWI ≤ 1.75
Sex	Men	Mean	3.389	2.987
		Predictors	NWHI > 3.1 Increase in conflict > 4	NWHI ≤ 3.9 PHWI > 2.75
	Women	Mean	3.396	3.312
		Predictors	Increase in conflict > 2 Negative effects of teleworking > 4.625	NHWI ≤ 2.75 Age > 38.5 PWHI ≤ 3.25 Responsibility for dependents > 2.5
Group	Students	Mean	3.619	3.432
		Predictors	NHWI > 1.75 NWHI > 4.55 Eating habits > 4	NHWI ≤ 2.25 PWHI > 3.25 NWHI ≤ 2.3
	A&S	Mean	3.174	3.8
		Predictors	Increase in conflict > 2 Coexistence environment = with parents or with other people	Increase in conflict ≤ 2 Support from acquaintances > 2 Increase in conflict ≤ 1 Coexistence environment = with a partner and children; alone; with parents; with a partner (no children)
	T&R	Mean	2.85	3.48
		Predictors	NWHI > 3.9 Residence size ≤ 135 m^2^	NHWI ≤ 2.75 NWHI ≤ 3.675 PWHI > 3.75

In general terms, for the global sample, an increase in conflict and NWHI were exclusively responsible for negative affective states (NAS), while NHWI, PWHI, and age were exclusively responsible for positive affective states (PAS). Specifically, participants with the highest scores in NAS were those who perceived a certain level of increase in conflict (above 2) and a high negative impact of work on their personal lives (NWHI) (above 4.1). Conversely, participants with the highest levels of PAS were those with medium to low levels of NHWI (below 1.75), aged over 42.5 years, and with medium to high levels of PWHI (above 3.25).

Comparing the results by sex, for men NWHI and an increase in conflict were responsible for the highest scores in NAS; specifically, medium to high levels of NWHI (above 3.1) and high levels of increase in conflict (above 4). Meanwhile, PAS were characterized exclusively by NWHI and PHWI. Men with medium to low levels of NWHI (below 3.9) and medium to high levels of PHWI (above 2.75) exhibited high PAS.

For women, an increase in conflict and the negative effects of teleworking characterized NAS, while NHWI, age, PWHI and responsibility for dependents characterized PAS. Medium to high levels of an increase in conflict (above 2) and experiencing negative effects due to teleworking were the highest predictors for NAS. In relation to PAS, medium to low levels of NWHI (below 2.75), an age of over 38.5 years, and having responsibilities over 2.5 or more dependents, even with medium to low levels of PWHI (below 3.25), resulted in high PAS.

Finally, when comparing by group, NWHI and NHWI emerged as the main predictors for students' NAS and PAS and T&Rs' PAS, while an increase in conflict was the main predictor for A&S. NWHI was the main predictor for T&Rs' NAS, while NHWI was mainly responsible for the students' NAS.

Specifically, for students, NHWI split the sample down the lines of NAS and PAS. On the negative side, students with even relatively low to high levels of NHWI (above 1.75), combined with high NWHI (above 4.55) and a high impact on eating habits (above 4), were those with the highest levels of NAS. By contrast, students with medium to low levels of NHWI (below 2.25), medium to high levels of PWHI (above 3.25) and low levels of NWHI (below 2.3) generated the highest scores in PAS.

For A&S, perceptions of an increase in conflict above 2 and coexistence with parents or other people (not relatives) were responsible for the highest levels of NAS. With regard to PAS, a level of support from acquaintances above 2, very low levels of increase in conflict (below 1), and coexistence with a partner and children, living alone, with parents, or with a partner (no children) predicted the highest levels of PAS.

Finally, the main predictor for T&Rs' NAS score was NWHI, while NHWI was the main predictor for PAS, combined with NWHI and residence size. T&R with medium to high levels of NWHI (above 3.9) and living in residences smaller than 135 m^2^ were those with the highest NAS. On the other hand, medium to low levels of NHWI (below 2.75) and NWHI (below 3.675), combined with medium to high levels of PWHI (above 3.75), predicted the highest levels of PAS.

## Discussion and Conclusions

The aim of this research was to analyze the emotional impact of the pandemic on students, teachers, and administrative staff, and the extent to which the psychosocial aspects are able to characterize their affective states. In this sense, our research provides a global overview, analyzing the extent to which sociodemographic variables (Liang et al., 2020; Losada-Baltar et al., 2020; González-Tovar and Hernández-Rodríguez, 2021), the impact of COVID on the subjects' environment (Sandín et al., 2020), the psychosocial context of coexistence and perceived social support (Losada-Baltar et al., 2020; Sandín et al., 2020; Ogrodniczuk et al., 2021), characteristics related to the physical context during the quarantine (Sandín et al., 2020), labor conditions (Sandín et al., 2020; Zurlo et al., 2020). and the work-life balance (Bhumika., 2020; Wan Mohd Yunus, 2021) can characterize the emotional states of the subjects during the quarantine (Sandín et al., 2020; González-Tovar and Hernández-Rodríguez, 2021).

Results showed that, based on group, students were the ones who have suffered the most as a result of this situation. They were affected at a higher percentage by temporary employment regulation, higher scores in NWHI and NHWI, lower scores in PHWI, and the negative impacts of teleworking. Additionally, they reported higher mean scores in interpersonal conflict and worse scores with regard to negative affective states. The increase in conflicts in the students' community can be explained by various factors such as continued coexistence (Cao et al., 2020) and the number of people living together (Wang et al., 2020).

The Global Research Study and Trends (Schleicher, 2020) indicated that “the impact of the pandemic is keenly felt across the globe, especially by students […] COVID-19 has negatively impacted student engagement, it has affected the work/career readiness of students and that more students are falling behind on their studies” (p.18). Other studies indicate that the greater impact on the group of students can be explained by their increased fear of a possible impact on themselves and/or relevant people in their immediate environment, on a physical level (Sultana et al., 2021), a psychopathological level (Mechili et al., 2021), an emotional level (Tasso et al., 2021), regarding psychological inflexibility (Hernández-López et al., 2021), personal or family economic-labor uncertainty (Essadek and Rabeyron, 2020; Kassim et al., 2021) or a transfer of their place of residence (Díaz-Jiménez et al., 2020), as well as isolation or social distance caused by the pandemic (Abouk and Heydari, 2021). All of these factors would not be sufficiently compensated by the social support received in the face of severe stress (Hunt et al., 2021), as is also evidenced by our results, since in the case of students, this variable does not help to characterize either the NAS or the PAS.

With regard to the affective states, the results obtained in this study are in line with those obtained by other investigations carried out in the context of the pandemic in terms of sex and age. Specifically, negative affective states (NAS) describe a profile with a greater impact among the youngest (students) compared to the oldest participants (mainly T&R and A&S). In our sample, and specifically in the group of students, there was a greater impact due to temporary employment regulation and temporary contracts, and the majority perceived a reduction in their economic capacity compared to other groups, which, combined with an enhanced perception of family stress, would lead to increased negative emotions. Recent studies have indicated age as a factor related to the negative emotional impact, possibly due to its interaction with job insecurity during the crisis, which has particularly affected young people and women (Rodríguez-Rey et al., 2020).

In terms of the sex of the students, the greater extent of negative emotions in women is consistent with previous studies in this group (Browning et al., 2021) and can be explained by the greater relevance assigned to learning in their professional future (Aristovnik et al., 2020), greater emotional expressiveness and less tolerance of uncertainty (Sundarasen et al., 2020).

Regardless of the group to which they belong, women tend to experience more negative effects in comparison to men, as has also been verified in various studies on mental health in times of COVID-19 (Esteban-Gonzalo et al., 2020; Sandín et al., 2020; Cénat et al., 2021). Furthermore, in our sample, this coincides with the fact that most workers in the health sector were women. Studies such as Luo et al. (2020) establish that being of female sex, together with working in the health sector, was a risk factor for experiencing anxiety, depression, and other negative emotions during confinement.

In terms of work-life balance, results revealed that the pandemic caused greater negative emotional effects in the students (NWH and NHW) and lower levels of positive effects (PHW) in relation to the other two groups (A&S and T&R), which was consistent with previous studies that compared students with university staff (Odriozola-González et al., 2020; Kassim et al., 2021). Likewise, various studies emphasize the difficulties faced by this group in adjusting to uncertainty in the short and long term, and in particular the effects caused by a possible delay in completing their studies (Kassim et al., 2021) and the loss of relationships resulting in a lack of interpersonal communication (Andrews et al., 2020; Xiao, 2020), but also the positive effects related to social well-being, such as high satisfaction with life and an optimistic vision of the future (Lukács, 2021).

The impact upon basic processes such as eating and sleeping habits is present in all groups, but differs by sex, as the impacts are more intense among women, as pointed out in previous studies on university students (Kalkan Ugurlu et al., 2021) or specifically in the case of female students when examining body dissatisfaction (Nolan et al., 2010). The negative effects of sleeping habits on emotional states are related to its fundamental role in recovering energy (Scotta et al., 2020); correspondingly, altered sleeping habits are related to changes in lifestyle, unstable economic resources, and social distance (Li et al., 2020).

The effects of teleworking/remote learning presented high negative levels (discomfort, irritability, and impatience) due to the perception of greater personal demand placed upon students and T&R, but not in the case of A&S. This can be explained by the difficulty in adjusting to the new learning processes, the shift to a remote working method or unclear instructional parameters (Tasso et al., 2021). For this reason, the negative effects were lower in the A&S group and could be attributed to a relatively minor change in work style, using the same computer applications. The transfer of the work / study environment to a personal/family space facilitates an increase in conflicts, NWHI, and an impact on sleeping/resting and eating habits, as other recent studies with students have shown (Cao et al., 2020; Wang et al., 2020). All this indicates a complex interaction of multiple factors, but in a different way for each group under study.

Different predictive factors were observed for NAS and PAS, as well as differences based on sex and group. Although the main predictive variables in general terms were the WLB and the increase in conflict, other variables also emerged, such as age, the negative effects of teleworking, the impact on eating habits, the living environment, responsibility for dependents, social support, and the size of the residence. All these variables have been previously discussed, with the exception of the size of the residences. This variable is a predictive variable of the NAS in T&R. Although currently, no studies are foreseen for this group, the study by Kalkan Ugurlu et al. (2021) indicated that in the case of students, the size of the residence had no relevance on the emotional state, as was also the case for our sample of students.

### Limitations and Future Research

Although this research presents valuable information on the impact of the pandemic on the university community, it also comes up against some limitations that must be considered. Firstly, this research was carried out in a Spanish university. The university is the largest in the Catalonia region and one of the largest in Spain, the third oldest in the country, and is a public university with a face-to-face studying format. Generalization of the results to other types of higher education centers, such as those that provided online training before the pandemic or that are of a smaller size in terms of students, teachers, and administrative and services staff, may not be appropriate. Therefore, future research should also collect data in other centers, analyzing which results are common for certain variables and which are directly influenced by the particularities of the educational center.

Secondly, results cannot be generalized over time due to the exceptionality of this pandemic situation. Future research should be directed at the study of the effects of uncertainty on students and teachers, as they are the most affected groups, and should analyze the degree of adjustment to the changes induced by the pandemic in participants' ability to cope with stress. Contextual factors such as unemployment or the recovery of economic capacity, the effects of vaccination processes, improvement of social relations, etc., play an important role into the management of uncertainty.

Finally, the existence of unbalanced groups in the sample should be considered a limitation of the study, as it did not represent the real distribution of university groups. Nevertheless, the total responses received are sufficiently representative of the reference population studied.

### Practical Implications

The pandemic has had an impact upon the affective states of the members of the university community, especially negative states. In this sense, it is essential to promote and disseminate psychological care services within the university context. Although various universities offer this service, it is necessary to also provide it online, and to promote interpersonal proactive actions geared toward all university groups to counteract the social distance induced by the technological change caused by COVID (Tasso et al., 2021).

Scotta et al. (2020) proposes the creation of secure platforms that facilitate the social interaction of students as long as face-to-face attendance restrictions are in place. The UB psychological care unit provided psychological support to all members of the university community during the pandemic, addressing the effects of the confinement at a personal and relational level, in addition to problems with daily eating routines, sleeping, and work planning. Future studies should analyze the effect of these interventions on the affective states of recipients in comparison to the general university population.

Furthermore, as has been found by various studies, the use of digital tools for prolonged periods of time increases the likelihood that subjects will experience “ZOOM fatigue” (Wiederhold, 2020), that is, anxiety, tension, and exhaustion. Therefore, it is important to provide more specific training in teleworking, especially for newly hired teaching and administrative staff. Undoubtedly, the effects caused by the adjustment to an online training methodology have been particularly felt by students and T&R, which is why it is essential to be aware of their technological and connectivity needs at the start of the teaching activity. This will make it possible to identify situations of vulnerability, both at the level of technological knowledge and available resources. Díaz-Jiménez et al. (2020) establishes the need to develop a university policy adopting resources to the remote learning modality and the transparency of the information provided. One resource put into practice at the UB during the pandemic period was the “Connecta UB” program, which provided laptops and / or internet connections to students without sufficient financial resources. This initiative is based on the recommendation of UNESCO. (2020), which urges the implementation of measures to ensure that all students have access to online education, and in the case that they do not have the infrastructure, to temporarily lend these types of devices as well as access to the internet.

Finally, the results of our research showed that confinement caused an increase in personal and relational conflicts, which is why we consider it necessary to pay special attention to work-life balance and respect working hours, especially for T&R and students. As pointed out by Sofo et al. (2021), “it is necessary to find strategies to preserve physical and mental well-being while avoiding negative psychological effects during the COVID-19 lockdown” (p. 99).

## Data Availability Statement

The raw data supporting the conclusions of this article will be made available by the authors, without undue reservation.

## Ethics Statement

The Equality Unit of the University of Barcelona approved the questionnaire and its administration to the three collectives (teachers and researchers, administrative and services staff, and students), once it had been guaranteed by the University ethics commission that all the information collected was confidential and was subject to current data protection regulations. The participants provided their written informed consent to participate in this study.

## Author Contributions

MR and MY-B contributed to theory development, research design, data analyses, and discussion. MJ and MS contributed to theory development, research design, and discussion. All authors contributed to the article and approved the submitted version.

## Conflict of Interest

The authors declare that the research was conducted in the absence of any commercial or financial relationships that could be construed as a potential conflict of interest.

## Publisher's Note

All claims expressed in this article are solely those of the authors and do not necessarily represent those of their affiliated organizations, or those of the publisher, the editors and the reviewers. Any product that may be evaluated in this article, or claim that may be made by its manufacturer, is not guaranteed or endorsed by the publisher.
